# Infant dietary intake of yogurt and cheese and gastroenteritis at 1 year of age: The Japan Environment and Children’s Study

**DOI:** 10.1371/journal.pone.0223495

**Published:** 2019-10-07

**Authors:** Mari Nakamura, Kei Hamazaki, Kenta Matsumura, Haruka Kasamatsu, Akiko Tsuchida, Hidekuni Inadera, on behalf of the Japan Environment and Children’s Study Group

**Affiliations:** 1 Department of Public Health, Faculty of Medicine, University of Toyama, Toyama, Japan; 2 Toyama Regional Center for JECS, University of Toyama, Toyama, Japan; Instiuto Ramon y Cajal de Investigacion Sanitaria (IRYCIS), SPAIN

## Abstract

**Background:**

The important role played by intestinal bacterial flora in human health has recently attracted public attention worldwide. Although yogurt is thought to help in preventing the onset of gastroenteritis, this property has rarely been examined in epidemiological studies.

**Method:**

This study analyzed data obtained by the Japan Environment and Children’s Study. From a dataset of 103,062 pregnancies, 82,485 were selected for this analysis. Dietary intake of fermented foods (yogurt and cheese) in 1-year-old infants was assessed with a food frequency questionnaire. Parent-reported physician-diagnosed gastroenteritis in early childhood was determined from a questionnaire conducted when the child was 1 year old.

**Result:**

The incidence of gastroenteritis was significantly lower in infants who consumed yogurt ≥ 7 and 3–6 times/week than in infants who consumed yogurt < 1 time/week in crude models (n = 82,485) and after adjustment for covariates (adjusted odds ratio [95% confidence interval], 0.78 [0.70–0.86] versus 0.82 [0.76–0.89], respectively; n = 65,051). Frequency of weekly cheese consumption was not associated with the incidence of gastroenteritis.

**Conclusion:**

Consumption of yogurt, but not cheese, at 1 year of age was associated with a reduced risk of gastroenteritis. Further studies of this association, including interventional studies, are warranted.

## Introduction

Probiotics are thought to be strongly associated with microbiota, based on the theory of longevity proposed by Metchnikoff in 1907 that posits the ingestion of yogurt containing *Lactobacillus bulgaricus* establishes *Lactobacillus* flora in the intestine, thereby inhibiting the growth of “putrefactive” bacteria and preventing autointoxication [1].

Various studies have examined the benefits of probiotics in gastroenteritis, and several clinical studies have found that probiotics can shorten the duration of diarrhea [2–5]. A study conducted in the Republic of Korea demonstrated that, compared with placebo, *Bifidobacterium longum* and *Lactobacillus acidophilus* had strong anti-rotavirus activity and significantly shortened the duration of the symptoms without adverse events [6]. In a study in Finland of 40 patients with diarrhea, compared with placebo, *Lactobacillus reuteri* was associated with a smaller proportion of symptomatic patients on day 2 (26% vs 81%, p = 0.0005) and short treatment duration (1.7 days vs 2.9 days, p = 0.07) [7]. However, several randomized controlled trials (RCTs) showed that the frequency of diarrhea was slightly, but not significantly, lower in probiotic groups than in placebo groups [8–10]. As part of a cohort analysis of elderly individuals with proximal femur fractures, Mallina et al. [11] reported no preventive effect of a probiotic against *Clostridium difficile*-associated diarrhea. In a questionnaire survey on the use of probiotic supplements in infants aged 0–18 months and related factors (e.g., infant health) in Taiwan, Chen et al. [12] determined that use of probiotic supplements was positively associated with parents’ higher educational level, higher household income, and healthy lifestyle and that frequency of diarrhea was reduced to a greater extent in infants receiving probiotic between 0 and 6 months of age than in those receiving probiotics between 7 and 18 months and between 0 and 18 months.

Many previous RCTs have been performed with the aim of preventing exacerbation of gastroenteritis in inpatients. The benefits of probiotics were not consistently proven in these studies. This led us to design the present study, which sought to identify prophylactic measures for gastroenteritis in “children living an ordinary life” that use commonly available yogurt and cheese and could be easily implemented in typical households. Such prophylactic measures would be of considerable value in terms of health economics: they would reduce the costs associated with hospitalization and treatment (e.g., infusion) of children and prevent interruption to their parents’ socially productive activities.

Here, we analyzed data from the Japan Environment and Children’s Study (JECS) to determine whether there is an association between gastroenteritis and frequency of consumption of probiotics, namely, yogurt and cheese in infants at 1 year of age. Our results indicated that infant consumption of yogurt, but not cheese, at 1 year of age was associated with a reduced risk of gastroenteritis.

## Methods

### JECS population

**Fig 1:**
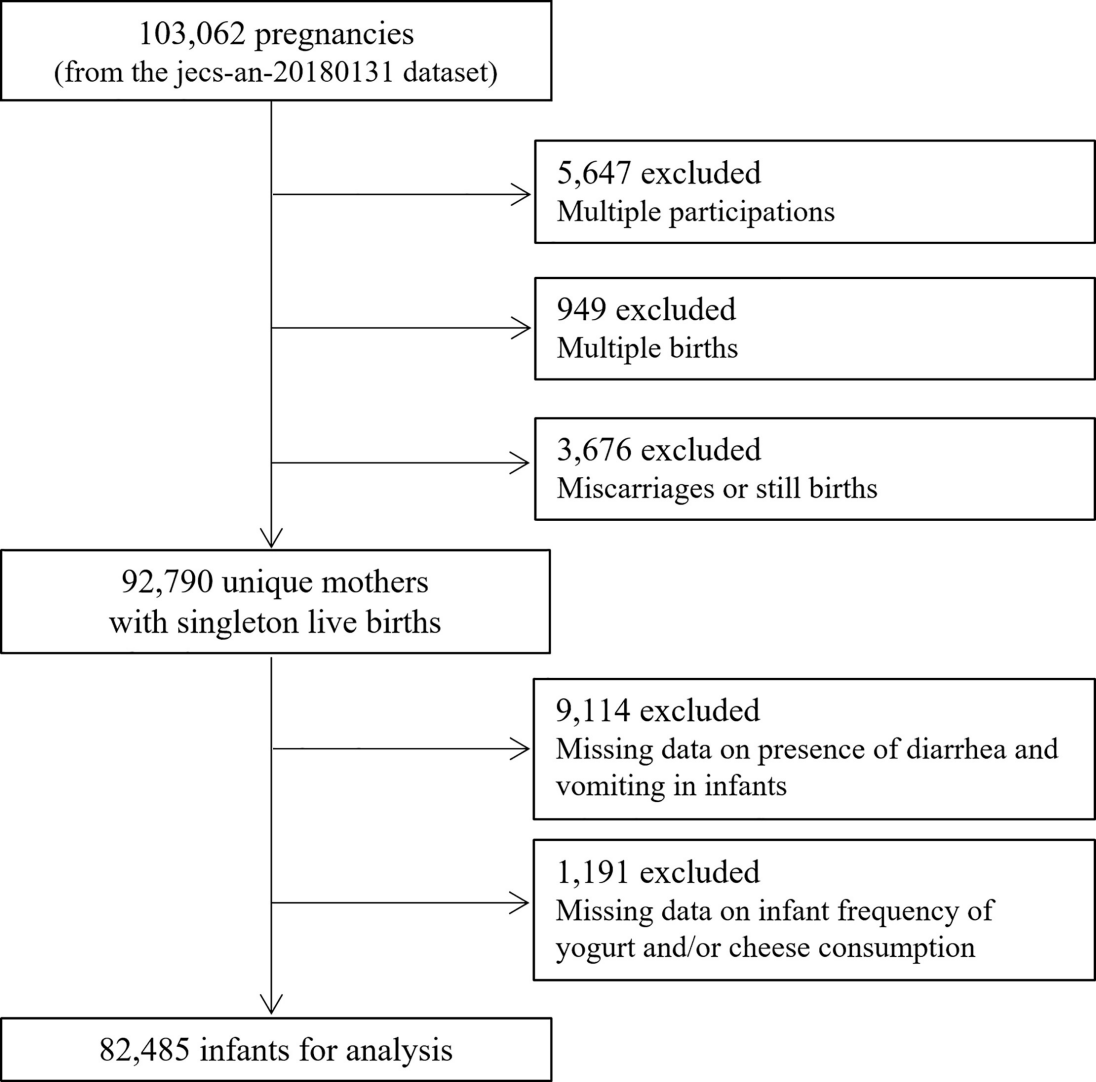
Flow diagram of the recruitment and exclusion process in this study.

This study was based on the JECS dataset for 103,062 pregnancies. The JECS is a nationwide, government-funded, multicenter, prospective birth cohort study proposed and launched by the Ministry of the Environment of Japan and lead by The National Center for JECS (program office), which was established in the National Institute for Environmental Studies; the study design has been described previously [13, 14] and the characteristics of the project population were reported by Michikawa et al. [14]. The goal of the JECS is to evaluate the effects of various environmental factors on children’s health and development. Pregnant women were recruited from 15 areas in Japan between January 2011 and March 2014. The present study is based on the jecs-an-20180131 dataset that was released in March 2018. A self-report questionnaire was administered to the mothers at first trimester, second/third trimester in pregnancy, 1 month after delivery, 6 months after delivery, and 1 year after delivery to collect demographics, medical and obstetric history, physical and mental health issues, lifestyle factors, occupation, and socioeconomic status. Fig 1 shows the recruitment and exclusion process in this study.

The study protocol was approved by the Institutional Review Board on Epidemiological Studies of the Japanese Ministry of the Environment, and the ethics committees of all participating institutions: the National Institute for Environmental Studies, National Center for Child Health and Development, Hokkaido University, Sapporo Medical University, Asahikawa Medical College, Japanese Red Cross Hokkaido College of Nursing, Tohoku University, Fukushima Medical University, Chiba University, Yokohama City University, University of Yamanashi, Shinshu University, University of Toyama, Nagoya City University, Kyoto University, Doshisha University, Osaka University, Osaka Medical Center and Research Institute for Maternal and Child Health, Hyogo College of Medicine, Tottori University, Kochi University, University of Occupational and Environmental Health, Kyushu University, Kumamoto University, University of Miyazaki, and University of the Ryukyus. All participants provided written informed consent. The JECS is conducted in accordance with the Helsinki Declaration and all other national regulations.

### Gastroenteritis

Data on gastroenteritis were collected 1 year after delivery via a self-administered questionnaire completed by mothers. Participants filled out 21 multiple choice check boxes referring to infants’ physician-diagnosed infectious diseases, one of which was “Vomiting and diarrhea (viral gastroenteritis, rotavirus, norovirus, and so on)”.

### Probiotics (yogurt and cheese)

To assess the frequency of probiotic intake, the following questions were included in the self-administered questionnaire at 1 year after delivery: “How many times a week does your child have yogurt?” and “How many times a week does your child have cheese?”. The response options were < 1 time/week, 1–2 days/week, 3–4 days/week, 5–6 days/week, 1 time/day, 2 times/day, and ≥ 3 times/day.

To estimate the incidence of gastroenteritis based on the intake of each probiotic, we categorized participant responses for the infant consumption of yogurt as < 1 time/week, 1–2 days/week, 3–6 days/week, or ≥ 7 times/week and cheese as < 1 time/week, 1–2 times/week, or ≥ 3 times/week.

### Confounding factors for multiple analyses

The confounding factors for multiple logistic models were recognized as follows: maternal age; number of previous deliveries (yes or no); body mass index (kg/m^2^) 1 month after delivery (< 18.5, 18.5–25, or ≥ 25); educational background (1. junior high school or high school, 2. technical junior college, technical/vocational college or associate degree, or 3. bachelor’s degree or postgraduate degree); annual household income (< 4 million JPY, 4–6 million JPY, or ≥ 6 million JPY); marriage status 6 months after delivery (married, divorced, widowed, or other); alcohol status 1 month after delivery (never, ex-drinker, 1–3 times/month, 1–3 times/week, 4–6 times/week, or every day); smoking status 1 month after delivery (never, quit before learning of pregnancy, quit after learning of pregnancy, currently smoking 10 cigarettes or less, currently smoking more than 10 cigarettes); physical activity during the second and third trimesters (METs-hour/week); employment status 1 year after delivery (employed or unemployed); birth weight (g); gestational age at birth (weeks); infant congenital abnormality (yes/no); communal life at 6 months of age (yes/no); consumption of breast milk at 1 year of age (yes/no); infant sex; caesarean delivery (yes/no); and age at which cow’s milk was introduced, including yogurt and cheese.

### Statistical analysis

Unless stated otherwise, data are expressed as mean ± standard deviation or median. Univariate and multivariate logistic analyses were applied to estimate the incidence of gastroenteritis. We calculated both unadjusted and adjusted odds ratios (ORs) with 95% confidence intervals (95% CIs). Data are expressed as the number of gastroenteritis cases (two groups: yes/no) for each category and proportion (%). All statistical analyses were performed by using SAS version 9.4 (SAS Institute Inc., Cary, NC). All two-sided p-values of less than 0.05 were considered statistically significant.

## Results

### Participants

Demographic and obstetric characteristics of participants (n = 82,485) are shown in Table 1. There tended to be more infants of multipara than nullipara. More cases had a mother who was employed than who was not, and more cases had experienced communal life at 6 months after birth than had not.

**Table 1:** Demographic and obstetric characteristics of participants (n = 82,485).

	Gastroenteritis			
	non-case		case	
	(n = 74,944)		(n = 7,541)	
**Maternal age, mean (SD)**	31.2	(5.0)	30.8	(5.0)
**Previous deliveries, n (%)**				
Nullipara	32,266	(44.2)	2,658	(36.1)
Multipara	40,776	(55.8)	4,715	(64.0)
**Pre-pregnancy BMI (kg/m**^**2**^**), n (%)**				
< 18.5	3,684	(5.1)	335	(4.7)
18.5–< 25	57,143	(79.8)	5,610	(77.9)
≥ 25	10,809	(15.1)	1,254	(17.4)
**Highest educational level, n (%)**				
Junior high school or high school	25,456	(34.4)	2,797	(37.5)
Technical junior college, technical/vocational college or associate degree	31,674	(42.8)	3,143	(42.2)
Bachelor’s degree, postgraduate degree	16,906	(22.8)	1,513	(20.3)
**Annual household income (JPY), n (%)**				
< 4 million	27,045	(39.0)	2,903	(41.4)
4–6 million	23,192	(33.5)	2,306	(32.9)
> 6 million	19,089	(27.5)	1,800	(25.7)
**Marital status, n (%)**				
Married (including common law marriage)	16,363	(98.2)	24,998	(98.1)
Divorced	136	(0.8)	217	(0.9)
Widowed	6	(0.04)	18	(0.1)
Others	167	(1.0)	252	(1.0)
**Alcohol intake, n (%)**				
Never	15,433	(91.5)	23,490	(90.8)
Ex-drinker	702	(4.2)	1,208	(4.7)
1–3 times / month	490	(2.9)	814	(3.2)
1–3 times / week	171	(1.0)	260	(1.0)
4–6 times / week	49	(0.3)	61	(0.2)
Every day	27	(0.2)	48	(0.2)
**Active smoking status, n (%)**				
Never	44,631	(60.1)	4,148	(55.6)
Quit before learning of pregnancy	16,707	(22.5)	1,773	(23.8)
Quit after learning of pregnancy	10,288	(13.9)	1,201	(16.1)
Currently smoking 10 cigarettes or less	2,136	(2.9)	256	(3.4)
Currently smoking more than 10 cigarettes	510	(0.7)	88	(1.2)
**Physical activity (mets h/ week), mean (SD)**	26.7	(56.3)	33.3	(70.4)
**Employed, n (%)**				
No	39,550	(53.6)	3,093	(41.5)
Yes	34,310	(46.5)	4,365	(58.5)
**Birth weight (g), mean (SD)**	3,023.4	(414.0)	3,038.5	(405.2)
**Gestational weeks, mean (SD)**	39.3	(1.5)	39.2	(1.5)
**Infants’ anomaly, n (%)**				
No	73,391	(97.9)	7,404	(98.2)
Yes	1,553	(2.1)	137	(1.8)
**Communal life 6 months after birth, n (%)**				
No	69,606	(94.3)	6,214	(83.9)
Yes	4,247	(5.8)	1,190	(16.1)
**Breastfeeding 1 year after birth, n (%)**				
No	28,848	(38.7)	3,743	(49.9)
Yes	45,634	(61.3)	3,755	(50.1)
**Infant’s sex, n (%)**				
Boy	38,249	(51.0)	4,090	(54.2)
Girl	36,695	(49.0)	3,451	(45.8)
**Cesarean delivery, n (%)**				
No	60,888	(81.5)	6,073	(80.7)
Yes	13,867	(18.6)	1,452	(19.3)
**Age at which cow’s milk was introduced, including yogurt and cheese, n (%)**				
≤ 6 months	6,608	(8.9)	618	(8.3)
7–8 months	27,978	(37.6)	2,687	(35.9)
9–10 months	23,324	(31.3)	2,338	(31.2)
11–12 months	9,932	(13.4)	1,171	(15.6)
≥ 13 months	882	(1.2)	117	(1.6)
Not yet	5,691	(7.7)	558	(7.5)

BMI: body mass index; SD: standard deviation

Cross tabulation analysis showed no clear relationship between frequency of yogurt consumption and that of cheese consumption (Table 2).

**Table 2:** Numbers of 1-year-old infants who consumed yogurt and cheese, n (%) (n = 82,485).

Cheese consumption						
	< 1 time/week		1–2 times/week		≥ 3 times/week	
**Yogurt consumption**						
< 1/week	12,277	(14.9)	3,946	(4.8)	813	(1.0)
1–2 times/week	11,802	(14.3)	12,344	(15.0)	1,953	(2.4)
3–6 times/week	9,282	(11.3)	11,067	(13.4)	4,655	(5.6)
≥ 7 times/week	5,475	(6.6)	5,612	(6.8)	3,259	(4.0)

ORs (95% CIs) for relationships between gastroenteritis and yogurt/cheese are shown in Table 3. The incidence of gastroenteritis was significantly lower among infants who consumed yogurt 3–6 times/week and ≥ 7 times/week. The frequency of cheese consumption did not have any significant effect on the incidence of gastroenteritis.

**Table 3:** Odds ratios (95% confidence intervals) for gastroenteritis according to frequency of infant consumption of yogurt and cheese at 1 year of age (n = 82,485).

Infant consumption	Case		Control	Crude OR (n = 82,485)	(95% CI)	*p*-value	Adjusted OR (n = 65,051)	(95% CI)	*p*-value
**Yogurt**									
< 1 time/week	1,678	/	15,358	reference			reference		
1–2 times/week	2,649	/	23,450	1.03	(0.97, 1.10)	0.310	0.93	(0.86, 1.00)	0.062
3–6 times/week	2,127	/	22,877	**0.85**	**(0.80, 0.91)**	**< 0.0001**	**0.82**	**(0.76, 0.89)**	**< 0.0001**
≥ 7 times/week	1,087	/	13,259	**0.75**	**(0.69, 0.81)**	**< 0.0001**	**0.78**	**(0.70, 0.86)**	**< 0.0001**
**Cheese**									
< 1 time/week	3,485	/	35,351	reference			reference		
1–2 times/week	3,157	/	29,812	**1.07**	**(1.02, 1.13)**	**0.006**	1.03	(0.97, 1.10)	0.300
≥ 3 times/week	899	/	9,781	0.93	(0.86, 1.01)	0.073	1.07	(0.98, 1.17)	0.162

Adjusted for maternal age, previous deliveries, body mass index, educational background, annual household income, marriage status, alcohol status, smoking status, physical activity, employment status, birth weight, gestational weeks, infant congenital anomaly, communal life, consumption of breast milk, infant sex, caesarean delivery, and age at which cow’s milk was introduced, including yogurt and cheese. Bold indicates significance (p < 0.05). CI: confidence interval; OR: odds ratio

## Discussion

In this large-scale cohort study, which is the first of its kind to investigate the relationship between consumption of fermented food and gastroenteritis in infants, the incidence of gastroenteritis was significantly lower in infants who consumed yogurt ≥ 7 times/week and 3–6 times/week. The questionnaire used in this study inquired about the frequency, but not the amount, of yogurt consumption, and the amount needs to be addressed in future work, for example, in a similar manner to a previous study that compared probiotic formulas with different concentrations of microorganisms [10].

We similarly examined another dairy product, cheese, in this study based on the assumption that it could have a similar effect to that of yogurt. However, there was no significant difference in the incidence of gastroenteritis according to differences in frequency of cheese consumption. A pertinent question is, therefore, whether the cheese consumed was actually probiotic because two types of cheese are available: one is natural cheese containing live lactic acid bacteria and enzymes, and the other is a processed cheese in which lactic acid bacteria are no longer alive due to heat treatment during the cheese-making process. The questionnaire used in the present study did not distinguish between these types of cheese, and this may be why a prophylactic effect of cheese was not observed. This issue needs to be addressed in future work.

Many previous RCTs failed to find significant effects of probiotics. Differences in food culture among the study countries and differences in bacterial strains might have contributed to the nonuniformity of results among studies. A study by Sur et al. [5] involving children aged 1–5 years in an urban slum in India found that probiotics could help to prevent acute diarrhea. In addition, in an RCT conducted in Vietnam by Hong Chau et al. [8], the time to the disappearance of diarrhea was shorter, albeit not significantly, in the group administered probiotics. On the other hand, Hatakka et al. [15] found no association between *lactobacillus rhamnosus* consumption and respiratory infection in children attending daycare centers in Finland. Conway et al. [9] compared gastroenteritis among groups without yogurt consumption, with ordinary yogurt consumption, and with bio-yogurt consumption and found no effect of yogurt consumption on gastroenteritis. The effect of yogurt consumption may be more apparent in Asian countries where yogurt is not a part of the traditional diet [16]. The present study is unique because a significant difference according to yogurt consumption was shown in more unrestricted conditions; for example, differences in bacterial strains in yogurt and differences in regional diet within Japan were not taken into consideration.

The strengths of the present study are that data on approximately 80,000 mother–child pairs were analyzed and therefore the results of such a large sample size adequately represent the maternal population in Japan. In addition, data were adjusted for a large number of possible covariates. Nonetheless, there are some limitations. Because a food frequency questionnaire was used, there may have been differences in the understanding of questions among responders. The food frequency questionnaire did not cover consumption of other probiotics and thus we could not assess their possible influence on the occurrence of gastroenteritis. Also, brands of yogurt and cheese products were not determined, so there were undoubtedly variations in bacterial strains. Furthermore, this is a cross-sectional study and, as such, causal relationships could not be identified.

In conclusion, this study showed a reduced risk of gastroenteritis in 1-year-old infants who consumed yogurt 3–6 times/week and ≥ 7 times/week. Based on these results, further work examining the relationship with microbiota and an interventional study are warranted.

## References

[1] Metchinikoff É. The Prolongation of Life, optomistic studies: The Knickerbocker Press; 1908.

[2] Fox MJA, K.D.K. Robertson L.K. Ball M.J. Eri R.D. Can probiotic yogurt prevent diarrhoea in children on antibiotics? A double-blind,randomised,placebo-controlled study. BMJ open. 2015;5(e006474). 10.1136/bmjopen-2014-006474 25588782 PMC4298112

[3] Binns CW, Lee AH, Harding H, Gracey M, Barclay DV. The CUPDAY Study: prebiotic-probiotic milk product in 1-3-year-old children attending childcare centres. Acta Paediatr. 2007;96(11):1646–50. Epub 2007/10/17. 10.1111/j.1651-2227.2007.00508.x . 17937689

[4] Szajewska H, Kotowska M, Mrukowicz JZ, Armanska M, Mikolajczyk W. Efficacy of Lactobacillus GG in prevention of nosocomial diarrhea in infants. J Pediatr. 2001;138(3):361–5. Epub 2001/03/10. 10.1067/mpd.2001.111321 . 11241043

[5] Sur D, Manna B, Niyogi SK, Ramamurthy T, Palit A, Nomoto K, et al Role of probiotic in preventing acute diarrhoea in children: a community-based, randomized, double-blind placebo-controlled field trial in an urban slum. Epidemiol Infect. 2011;139(6):919–26. Epub 2010/07/31. 10.1017/S0950268810001780 . 20670468

[6] Lee DK, Park JE, Kim MJ, Seo JG, Lee JH, Ha NJ. Probiotic bacteria, B. longum and L. acidophilus inhibit infection by rotavirus in vitro and decrease the duration of diarrhea in pediatric patients. Clin Res Hepatol Gastroenterol. 2015;39(2):237–44. Epub 2014/12/03. 10.1016/j.clinre.2014.09.006 . 25459995

[7] Shornikova AVC, I.A. Isolauri E. Mykkanen H. Vesikari T. *Lactobacillus reuteri* as a Therapeutic Agent in Acute Diarrhea in Young Children. Journal of Pediatric Gastroenterology and Nutrition. 1997;24(4):399–404. 10.1097/00005176-199704000-00008 9144122

[8] Hong Chau TT, Minh Chau NN, Hoang Le NT, Chung The H, Voong Vinh P, Nguyen To NT, et al A Double-blind, Randomized, Placebo-controlled Trial of Lactobacillus acidophilus for the Treatment of Acute Watery Diarrhea in Vietnamese Children. Pediatr Infect Dis J. 2018;37(1):35–42. Epub 2017/08/09. 10.1097/INF.0000000000001712 28787388 PMC5681247

[9] Conway S, Hart A, Clark A, Harvey I. Does eating yogurt prevent antibiotic-associated diarrhoea? A placebo-controlled randomised controlled trial in general practice. Br J Gen Pract. 2007;57(545):953–9. Epub 2008/02/07. 10.3399/096016407782604811 18252070 PMC2084134

[10] Mao M, Yu T, Xiong Y, Wang Z, Liu H, Gotteland M, et al Effect of a lactose-free milk formula supplemented with *bifidobacteria* and *streptococci* on the recovery from acute diarrhoea. Asia Pac J Clin Nutr. 2008;17(1):30–4. Epub 2008/03/28. . 18364323

[11] Mallina R, Craik J, Briffa N, Ahluwalia V, Clarke J, Cobb AG. Probiotic containing *Lactobacillus casei*, *Lactobacillus bulgaricus*, and *Streptococcus thermophiles* (ACTIMEL) for the prevention of Clostridium difficile associated diarrhoea in the elderly with proximal femur fractures. J Infect Public Health. 2018;11(1):85–8. Epub 2017/06/28. 10.1016/j.jiph.2017.04.001 . 28652125

[12] Chen YC, Chien YW, Chang PJ, Hsieh WS, Chen PC. Probiotic supplement use among young children in Taiwan: a prospective cohort study. PLoS One. 2012;7(9):e43885 Epub 2012/09/18. 10.1371/journal.pone.0043885 22984450 PMC3440429

[13] Kawamoto T, Nitta H, Murata K, Toda E, Tsukamoto N, Hasegawa M, et al Rationale and study design of the Japan environment and children's study (JECS). BMC Public Health. 2014;14:25 Epub 2014/01/15. 10.1186/1471-2458-14-25 24410977 PMC3893509

[14] Michikawa T, Nitta H, Nakayama SF, Ono M, Yonemoto J, Tamura K, et al The Japan Environment and Children's Study (JECS): A Preliminary Report on Selected Characteristics of Approximately 10 000 Pregnant Women Recruited During the First Year of the Study. J Epidemiol. 2015;25(6):452–8. Epub 2015/04/29. 10.2188/jea.JE20140186 25912098 PMC4444500

[15] Hatakka K, Savilahti E, Ponka A, Meurman JH, Poussa T, Nase L, et al Effect of long term consumption of probiotic milk on infections in children attending day care centres: double blind, randomised trial. BMJ. 2001;322(7298):1327 Epub 2001/06/02. 10.1136/bmj.322.7298.1327 11387176 PMC32161

[16] Market Trends in Taiwanese daily-daily industry and milk -daily products [Internet]. 1997. Available from: http://lin.alic.go.jp/alic/month/fore/1997/mar/spe-01.htm.

